# Case Report: Lymphatic malformation in a paediatric patient with a 7-year follow-Up

**DOI:** 10.3389/fped.2026.1836317

**Published:** 2026-05-29

**Authors:** Luyao Wang, Lei Guo

**Affiliations:** 1 Department of Vascular Anomalies and Interventional Radiology, Children's Hospital Affiliated to Shandong University, Jinan, China; 2 Department of Vascular Anomalies and Interventional Radiology, Jinan Children's Hospital, Jinan, China

**Keywords:** lymphatic malformation, haemorrhagic, long follow-up, pediatric, sclerotherapy

## Abstract

Lymphatic malformations (LMs) are common slow-flow vascular anomalies in children. Macrocystic LMs are particularly prone to haemorrhage and may result in life-threatening airway compression. This report describes a 2-month-old female infant with a mixed lymphatic malformation involving the maxillofacial and cervical regions complicated by acute intracystic haemorrhage and severe dyspnoea. Initial management administered at an external institution simple cyst aspiration without sclerotherapy, with subsequent rapid disease progression. The patient subsequently underwent emergency sclerotherapy with pingyangmycin (PYM, also known as bleomycin A5) under combined ultrasound and digital subtraction angiography guidance, followed by prolonged oral sirolimus therapy for 2 years. Over a 7-year follow-up period, the lesions demonstrated a durable response without significant recurrence. This case highlights that single session interventional sclerotherapy combined with adjunctive sirolimus therapy can achieve excellent long term outcomes in infants with life-threatening mixed LMs. These findings provide valuable information for the clinical management of this severe condition.

## 1 Introduction

Lymphatic malformations (LMs) are benign cystic lesions resulting from developmental anomalies of the lymphatic system (1). According to the International Society for the Study of Vascular Anomalies (ISSVA), LMs are classified as slow-flow vascular anomalies and predominantly occur in the head and neck region (2). Radiologically, they are categorised as macrocystic (locular volume ≥ 2 cm^3^), microcystic (< 2 cm^3^), or mixed type (3).

When complicated by intracystic haemorrhage, LMs can rapidly expand, leading to compression of adjacent structures and impairment of vital functions such as breathing and swallowing, with the potential to become life-threatening (4, 5). This case report describes a 2-month-old infant with a life-threatening hemorrhagic macrocystic LMs who was successfully treated with single session interventional sclerotherapy combined with sirolimus therapy, with a 7-year long-term follow-up. This report aims to provide clinical evidence supporting for the safety and effectiveness of this treatment strategy for severe infantile LMs.

## 2 Case description

### 2.1 Patient presentation

A 2-month-old female infant was referred to our hospital with progressive dyspnoea, sublingual haemorrhage, and an enlarging orofacial mass. The mass had been noted since birth, resulting in facial asymmetry and limited tongue mobility. Both parents were healthy, with no family history of hereditary or congenital disorders.

Four days prior to admission, the infant underwent simple cyst aspiration at another institution. Approximately 7 mL of light-red fluid was aspirated and no sclerotherapy was administered. Post-intervention, the mass rapidly enlarged and became firm, accompanied by worsening dyspnoea and persistent sublingual oozing.

### 2.2 Physical examination

Physical examination revealed generalised skin pallor. A diffuse, firm, and poorly mobile mass was palpable in the right maxillofacial and cervical region. Continuous, non-pulsatile bleeding was observed in the oral cavity. The tongue appeared markedly swollen, purplish, and rigid (Figure A).

### 2.3 Laboratory examination

Laboratory testing revealed a haemoglobin level of 67 g/L (reference range, 110–170 g/L),consistent with haemorrhagic anaemia. The mean corpuscular haemoglobin was 28pg (reference range, 27–31pg), which was within normal limits.

### 2.4 Imaging investigations

Preoperative magnetic resonance imaging (MRI) demonstrated a large, multiloculated, and diffusely infiltrative cystic lesion in the right orofacial and cervical region, measuring approximately 99.6 mm × 80.1 mm × 95.4 mm. The lesion caused severe airway narrowing and leftward (contralateral) deviation of the airway (Figures B–C).

**Figure 1:**
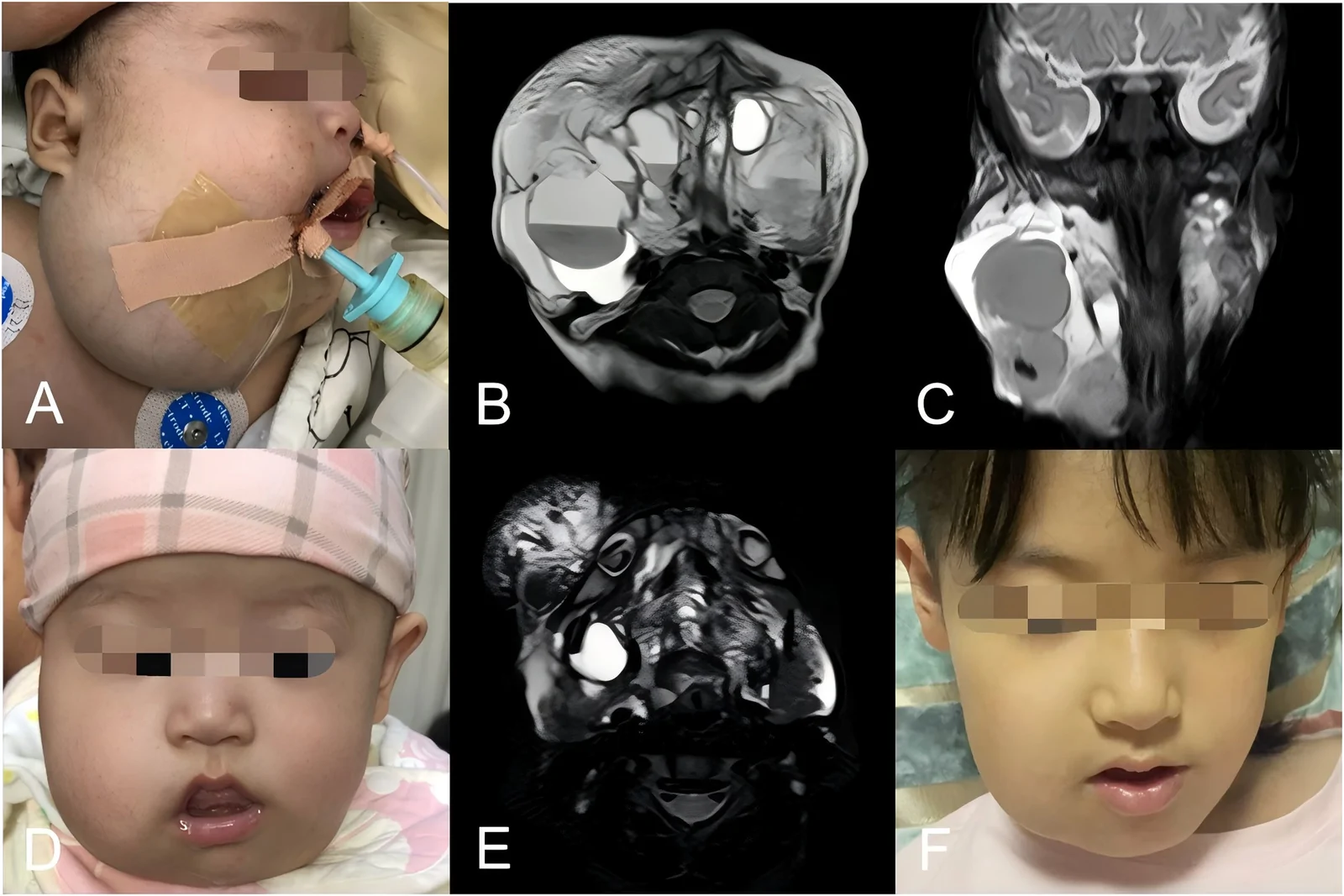
**(A)** A preoperative photograph demonstrated diffuse swelling of the maxillofacial and cervical regions, with non - pulsatile oral haemorrhage. **(B–C)** Preoperative axial and coronal MRI sequences demonstrated extensive abnormal signal intensities in the subcutaneous tissue, tongue, sublingual, submental, and cervical regions. The lesion presented as multiloculated cysts of varying sizes, with focal fluid - fluid levels. This led to a marked narrowing of the airway, with a significantly reduced cross - sectional area, and a leftward (contralateral) deviation of the airway from its normal tubular. **(D–E)** 3-month follow-up postmanagement photograph showed a significant reduction of the size of the massive maxillofacial and cervical swelling. MRI revealed remarkable improvement of the orofacial lymphatic malformation compared with before. There was no evidence of recurrence or hemorrhage. **(F)** At the 7-year follow-up: Clinical photograph showed persistent clinical stable.

Ultrasonography revealed a multicystic lesion with well-defined septations in the maxillofacial region. Multiple hypoechoic nodules were palpated in the left cervical region, with the largest measured approximately 1.4 × 0.6 cm and exhibited clear margins and homogeneous internal echoes.

According to ISSVA classification, a diagnosis of mixed LMs complicated by intracystic haemorrhage was established.

### 2.5 Intervention

Following a multidisciplinary evaluation, emergency airway management and supportive care were administered. Under combined ultrasonography and digital subtraction angiography (DSA) guidance, approximately 35 mL of purplish-red, turbid, non coagulable fluid was aspirated. Two 5-French drainage catheters were subsequently placed into the two largest cystic compartments to alleviate mass effect.

Sclerotherapy was then performed on this 5 kg infant using a mixture containing 1 mg PYM and contrast medium to enable fluoroscopic visualisation. Before completion of the procedure, single frame DSA image was acquired under DSA fluoroscopy and compared with preoperative MRI to ensure complete coverage of all cystic components and to prevent missed lesions, thereby optimising therapeutic efficacy (6).

### 2.6 Outcomes

MRI examinations were performed at the 3-month follow-up (Figure D-E) and periodically thereafter, MRI revealed remarkable improvement of the orofacial LMs, with minimal residual fibrosis and no evidence of recurrence or further haemorrhage. Image restoration analysis was not performed, as the patient demonstrated excellent clinical recovery and favourable long- term outcomes.

### 2.7 Follow-up

Oral sirolimus therapy was initiated following discharge and continued for 2 years with a target trough concentration of 5–15 ng/mL. The total follow-up period was 7 years (Figure F). At 4 years post-intervention, the patient experienced a small focal haemorrhage and received pingyangmycin injection in the outpatient department, without any obvious clinical complications during this period.

### 2.8 Ethical approval

This study was reviewed and approved by the Institutional Review Board of the Hospital Affiliated to Shandong University (Jinan Children's Hospital). Written informed consent was obtained from the patient's legal guardians for participation and for the publication of any potentially identifiable images or clinical data.

## 3 Discussion

LMs are usually typically identified at birth and usually manifest as a soft-to-firm masses with intact overlying skin. Morphologically, LMs are classified into macrocystic, microcystic, and combined types (7). Intracystic haemorrhage is a common phenomenon in LMs. Sclerotherapy is currently the standard first-line treatment for LMs at present (8, 9), and sirolimus has also demonstrated remarkable therapeutic effects (10). Ultrasound guidance is widely used for lymphatic malformation surgery,however, previous studies have shown that combining sclerosant with contrast medium under single-frame DSA fluoroscopy and ultrasound guidance can ensure safety and effectiveness, reduce the number of punctures and shorten hospital stay (6).

In the present case, this combined imaging-guided approach was employed using single-frame DSA fluoroscopy. This technique provides more comprehensive lesion visualisation, reduces the need for repeated interventional sessions, and minimises radiation exposure, supporting its clinical applicability. Although short-term outcomes of this approach have been reported previously (11), the extended follow-up in the present study confirms its long-term safety and efficacy when combined with adjunctive sirolimus therapy. Furthermore, based on the experience from this case and a review of previously reported cases (12), simple aspiration alone, without sclerosing agent injection, is not recommended during treatment, and an individualised clinical plan must be developed.Therefore, how to prevent, intervene in advance, and provide immediate treatment is crucial and serves as the guiding principle of current clinical practice.

This study is limited by its retrospective, single-case design. Findings from a single centre may not be generalisable to a broader population. In addition, large-scale, controlled studies with long-term follow-up are needed to further establish the optimal treatment strategy and to better define the long-term safety and efficacy of combined interventional and pharmacological therapies for extensive LMs complicated by acute haemorrhage in infants.

## 4 Patient perspective

The guardians were satisfied with clinical efficacy and committed themselves to regular long-term follow-up.

## Data Availability

The datasets presented in this article are not readily available because of ethical and privacy restrictions. Requests to access the datasets should be directed to the corresponding author.
