# Correction: Case Report: Lymphatic malformation in a paediatric patient with a 7-year follow-up

**DOI:** 10.3389/fped.2026.1905484

**Published:** 2026-09-14

**Authors:** Luyao Wang, Lei Guo

**Affiliations:** 1 Department of Vascular Anomalies and Interventional Radiology, Children’s Hospital Affiliated to Shandong University, Jinan, China; 2 Department of Vascular Anomalies and Interventional Radiology, Jinan Children’s Hospital, Jinan, China

**Keywords:** lymphatic malformation, haemorrhagic, long follow-up, pediatric, sclerotherapy

Author affiliation

Adding:

Affiliation Department of Vascular anomalies and Interventional Radiology, Jinan Children's Hospital, Jinan, China was omitted from the paper. This affiliation has now been added for author(s) Luyao Wang and Lei Guo.

Affiliation Department of Vascular anomalies and Interventional Radiology, Children's Hospital Affiliated to Shandong University, Jinan, China was erroneously given as Department of Intervention and Hemangioma, Children's Hospital Affiliated to Shandong University, Jinan, Shandong Province, China.

Error in figure/table

Wrong content

There was a mistake in Figure 1 and Figure 2 as published.

A correction has been made to the figures. The original FIGURE 1 and FIGURE 2 are removed entirely. Six out of the total twelve subpanels from the two original figures are selected and rearranged into a new figure designated as Figure.

Subpanels of the new figure are reorganized into groups (A), (B–C), (D–E) and (F) with separate legends. The legend for the newly grouped (B–C) is adapted from the original legend for the (A–C) group in FIGURE 1, with the text from “normal tubular” onwards omitted, while legends for (A), (D–E) and (F) are revised to match the updated grouping.

The corrected figure and caption are below.

**Figure 1:**
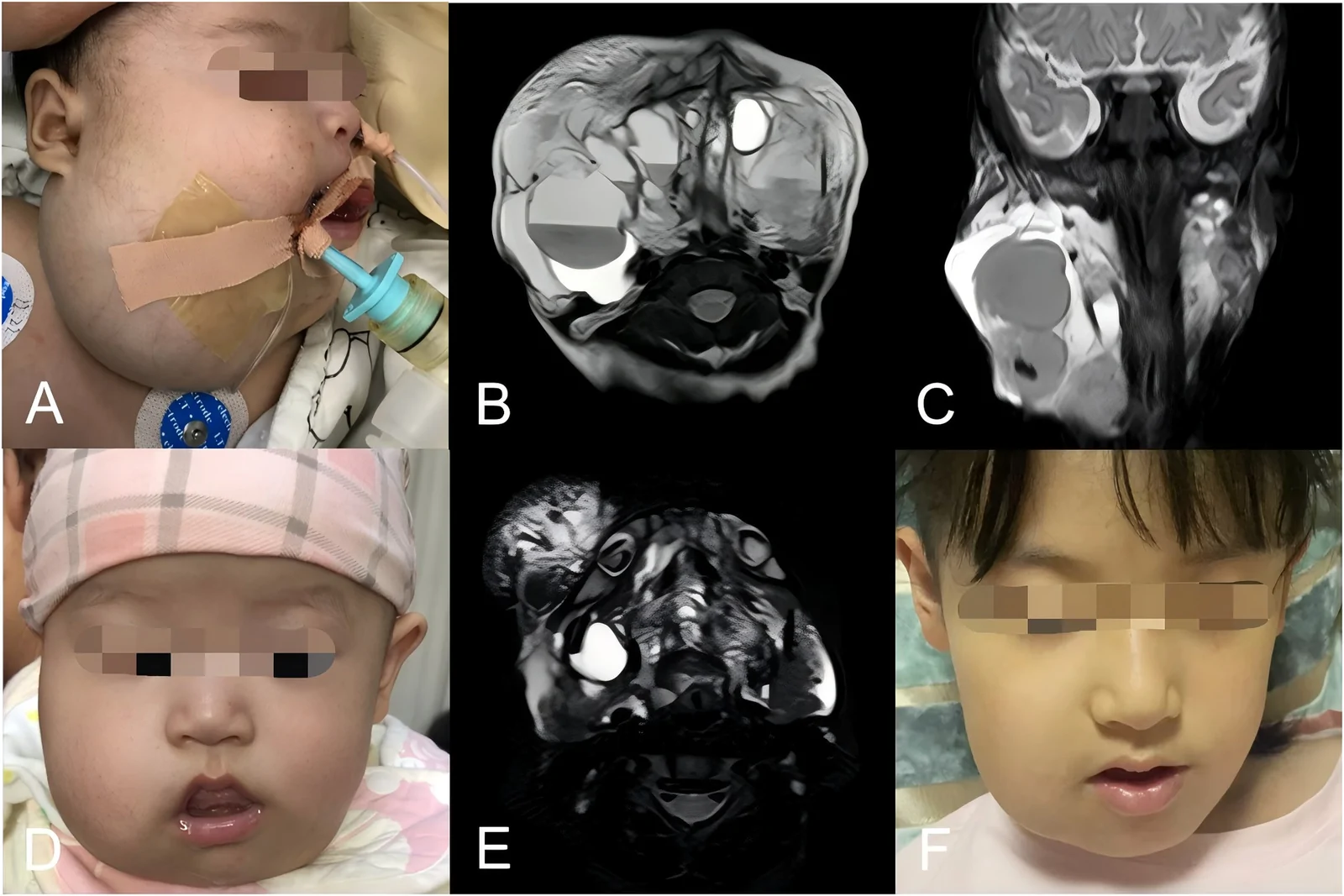


The new figure is organized into four groups, with legends as follows:

“Figure 1 (A) A preoperative photograph demonstrated diffuse swelling of the maxillofacial and cervical regions, with non—pulsatile oral haemorrhage. (B–C) Preoperative axial and coronal MRI sequences demonstrated extensive abnormal signal intensities in the subcutaneous tissue, tongue, sublingual, submental, and cervical regions. The lesion presented as multiloculated cysts of varying sizes, with focal fluid—fluid levels. This led to a marked narrowing of the airway, with a significantly reduced cross—sectional area, and a leftward (contralateral) deviation of the airway from its normal tubular. (D–E) 3-month follow-up postmanagement photograph showed a significant reduction of the size of the massive maxillofacial and cervical swelling. MRI revealed remarkable improvement of the orofacial lymphatic malformation compared with before. There was no evidence of recurrence or hemorrhage. (F) At the 7-year follow-up: Clinical photograph showed persistent clinical stable.

In the abstract, “Initial management at an external institution simple cyst aspiration without sclerotherapy, which led to rapid disease progression.” has been corrected to read:

“Initial management administered at an external institution simple cyst aspiration without sclerotherapy, with subsequent rapid disease progression.”

In the abstract, “Lymphatic malformations (LMs) are common low-flow vascular anomalies in children.” has been corrected to read “Lymphatic malformations (LMs) are common slow-flow vascular anomalies in children”

The label “Figure A” was omitted at the end of Paragraph 1 of Section 2.2 Physical examination.

A correction has been made to the section Section2.2 Physical examination, Paragraph 1: The label “Figure A” was omitted.

The corrected paragraph should read:

“The tongue appeared markedly swollen, purplish, and rigid (Figure A)”

Corrections have been made to Section 2.4, Imaging investigations, Paragraph 1, Paragraph 2, and Paragraph 3.

The corrected text is as follows:

For Paragraph 1: “The lesion caused severe airway narrowing and leftward (contralateral)deviation of the airway(Figure B–C)”

For Paragraph 2: “ and exhibited clear margins and homogeneous internal echoes”

For Paragraph 3: “According to ISSVA classification, a diagnosis of mixed LMs complicated by intracystic haemorrhage was established.”

The label “Figure 1E, F” was erroneously included at the end of Paragraph 2 of Section 2.5 Intervention.

A correction has been made to the section 2.5 Intervention, Paragraph 2:

The corrected labels are as follows:

For Paragraph 2: Sclerotherapy was then performed on this 5 kg infant using a mixture containing 1 mg PYM and contrast medium to enable fluoroscopic visualisation. Before completion of the procedure, single frame DSA image was acquired under DSA fluoroscopy and compared with preoperative MRI to ensure complete coverage of all cystic components and to prevent missed lesions, thereby optimising therapeutic efficacy (6).

A correction has been made to the the manuscript to address several issues. A correction has been made to the manuscript to address several issues. Specifically, the contents and headings of Sections 2.6 and 2.7 have been swapped, and the heading of Section 2.7 has been updated to “Follow-up.” Missing figure citations have been added, and the text in Section 2.6 has been revised by deleting a redundant phrase, an inaccurate word, and the figure citation “(Figure 2)” at the end of the section.

For the new Section 2.6:

“Outcomes

MRI examinations were performed at the 3-month follow-up (Figure D-E) and periodically thereafter, MRI revealed remarkable improvement of the orofacial LMs, with minimal residual fibrosis and no evidence of recurrence or further haemorrhage. Image restoration analysis was not performed, as the patient demonstrated excellent clinical recovery and favourable long- term outcomes.”

For the new Section2.7:

“follow-up

Oral sirolimus therapy was initiated following discharge and continued for 2 years with a target trough concentration of 5-15 ng/mL. The total follow-up period was 7 years (Figure F). At 4 years post-intervention, the patient experienced a small focal haemorrhage and received pingyangmycin injection in the outpatient department, without any obvious clinical complications during this period.”

The error that was made is in Section 2.8, Ethical Approval, where the hospital's affiliation was incorrectly stated as “hospital of Shandong University, Jinan, China”. This contains two errors: the preposition “of” is not the most accurate term for affiliation, and the location “Jinan, China” is error.

A correction has been made to the section 2.8, Ethical approval:

“..Board of the Hospital Affiliated to Shandong University (Jinan Children's Hospital).”

The term “slow flow” was incorrectly written as “low flow” in two separate locations within the manuscript.

A correction has been made to the opening paragraph preceding the Introduction section and to the first paragraph of the Introduction section itself.

The incorrect text “low-flow” has been replaced with the correct term “slow- flow” in both instances.

State the error that was made: In the second paragraph of the Introduction and the discussion, the abbreviation for “Lymphatic Malformations” was incorrectly written as “LM”.

A correction has been made to the second paragraph of the Introduction: The incorrect abbreviation “LM” has been corrected to “LMs”.

The correct wording is: “hemorrhagic macrocystic LMs”

A correction has been made to the last paragraph of the Discussion: The incorrect abbreviation “LM” has been corrected to “LMs”.

The correct wording is: “pharmacological therapies for extensive LMs complicated by acute haemorrhage in infants.”

The original version of this article has been updated.

